# An exploratory semantic analysis of age-related stereotypes in OpenAI’s GPT 4o model

**DOI:** 10.1093/geront/gnaf291

**Published:** 2025-12-08

**Authors:** Wan Hong, Moon Choi

**Affiliations:** Graduate School of Science and Technology Policy, Korea Advanced Institute of Science and Technology (KAIST), Daejeon, Republic of Korea; Graduate School of Science and Technology Policy, Korea Advanced Institute of Science and Technology (KAIST), Daejeon, Republic of Korea; Graduate School of Data Science, Korea Advanced Institute of Science and Technology (KAIST), Daejeon, Republic of Korea

**Keywords:** Ageism, Generative artificial intelligence, Large language model, Bias

## Abstract

**Background and Objectives:**

Generative artificial intelligence, particularly large language models (LLMs), is increasingly used to navigate information, potentially shaping users’ perceptions of different social groups. This study examines age-related stereotypes in LLM-generated text using natural language processing (NLP) techniques.

**Research Design and Methods:**

To ensure neutrality, extensive pilot testing was conducted to craft a prompt that did not elicit bias yet generated coherent responses. The final prompt, “Describe the personality of a [AGE]-year-old person,” was used with OpenAI’s GPT-4o API in February 2025, varying AGE from 10 to 90 in 10-year increments. The analysis was guided by the Stereotype Content Model, which assesses social cognition along two key dimensions: warmth (sociability, morality) and competence (ability, assertiveness). Scores were quantified using sentence embeddings.

**Results:**

Text similarity and stereotype content analyses revealed three age clusters, with older adults showing the most internal consistency. Descriptions of individuals aged 60 years and above featured relatively higher warmth but lower competence compared to younger groups. Notably, positive assertiveness terms were rarely used to describe older adults.

**Discussion and Implications:**

Findings suggest that GPT-4o may embed subtle age-related stereotypes, even when using largely positive language. These patterns potentially influence user perceptions through repeated exposure. Future research should investigate the mechanisms behind these biases and explore mitigation strategies to promote more age-inclusive artificial intelligence–generated content.

Today, generative artificial intelligence (AI) is one of the most influential pieces of emerging technology. Particularly, large language models (LLMs) have experienced a dramatic increase in performance and are now able to generate text similar in quality to human-generated text. LLMs hold the potential to transform communication, access to information, and even the way individuals perceive and interact with the world. A wide range of applications of LLMs are currently being explored, including customer service, education, content generation, healthcare, and gaming (Bansal et al., 2024). OpenAI’s GPT models are considered one of the forerunners of LLM technology, with capabilities ranging from answering simple questions to writing functioning computer programs. With a 65% global awareness (Maslej et al., 2024) and 400 million weekly active users (Rooney, 2025), ChatGPT, OpenAI’s web interface service for their language models, is regarded as synonymous with LLMs.

As is the case with other LLMs, OpenAI’s GPT models are large-scale statistical models that assign probabilities to word sequences. These probability values are used to predict the most likely sequence of words that would follow a given word or phrase, thereby generating a natural flow of text (Brants et al., 2007). The probabilities for word sequences are obtained by analyzing massive amounts of text, which are either publicly available or crawled from the internet using bots. Consequently, the texts generated by LLMs reflect the beliefs and perceptions of various social groups and carry the risk of propagating existing stereotypes back to society. Previous research has shown that people are more likely to seek advice from digital assistants on private or sensitive issues (Wester et al., 2024) and that even experts doing text annotation are influenced by suggestions from large language models (Choi et al., 2024).

A plethora of studies have identified and proposed methods to mitigate embedded stereotypes in LLMs, especially in relation to gender and race (Kotek et al., 2023; Lim & Pérez-Ortiz, 2024). Some methods assess LLM output characteristics, such as toxicity and sentiment, in an automated manner (Huang et al., 2020). Other methods use crowd-sourced judgements of harmfulness to identify problematic outputs (Thoppilan et al., 2022). However, there are concerns that even though these mitigation methods filter out explicitly toxic or harmful content, subtle stereotypes toward marginalized groups still exist (Gadiraju et al., 2023). If left unchecked, these subtle stereotypes may, over time, shape a user’s beliefs and perceptions of different social groups through incidental learning (Zukin & Snyder, 1984). The absence of attention to ageism and age-related stereotypes in mainstream discourse of AI bias risks another form of “digital ageism” propagated and maintained by AI (Chu et al., 2022).

## Age-related stereotypes

Stereotypes are pervasive in society, shaping perceptions, interactions, and policy decisions. Stereotypes are formed from homogenization of out-groups, especially in ways that highlight and heighten the differences between the in-group and out-group (Judd & Park, 1988; Taylor et al., 1978). The highlighted attributes to categorize different behaviors are then linked to assumptions about specific groups (Hewstone, 1983). Among these, age-related stereotypes play a significant role in influencing how older adults are perceived and treated. Classic gerontology research has identified some of the most common myths associated with aging, for example, being prone to sickness, lacking the ability to learn new things, being too late to adopt healthy lifestyles, having a low sex drive and sexual performance, and being dependent on society (Rowe & Khan, 1997). Other common negative stereotypes include older adults being frail, weak, grumpy, useless, and lonely (Palmore, 1999). This type of negative representation is present in various media, including movies (Lauzen & Dozier, 2005; Robinson et al., 2007), television shows (Markov & Yoon, 2021), and social media groups (Levy et al., 2014). Positive stereotypes of older adults portray them as being kind, wise, dependable, and having political influence (Palmore, 1999). These age-related stereotypes affect older adults in more ways than just influencing societal perceptions or decisions. Studies have shown that personal experiences of ageism or negative age-related stereotypes lead to decreased small motor control (Levy & Leifheit-Limson, 2009), cognitive performance (Hehman & Bugental, 2015), and reduced adoption of digital technology (Köttl et al., 2021).

## Quantifying embedded stereotypes in large language models

Given the widespread presence of age-related stereotypes in media, it is crucial to examine how these biases are embedded in large language models, as their potential as foundational models for multimodal AI agents and conversational agents is being explored in many capacities (Bansal et al., 2024). It is therefore unsurprising that many previous studies have explored other stereotypes embedded in LLMs and their mitigation methods (Dong et al., 2024; Gallegos et al., 2025).

Stereotypes in large language models have primarily been measured by examining the probability that a model is expected to generate stereotypical content. Various methods have been studied to compare the probability differences between generating stereotypical and anti-stereotypical content given the start of a sentence (Barikeri et al., 2021; Yeo & Chen, 2020). Other methods include creating multiple-choice question tasks, with responses representing stereotypes, anti-stereotypes, and unrelated statements (Chen et al., 2025; Li et al., 2020; Nadeem et al., 2021). However, these methods may not accurately reflect the actual usage of LLMs in real-life scenarios. Most in-the-wild tasks involve open-ended text generation in response to a prompt, not choosing from among given answers to a question or completing sentences. Previous research has also shown that the absence of explicitly toxic outputs from LLMs does not necessarily mean that the generated text lacks stereotypes (Gadiraju et al., 2023).

## The stereotype content model

The Stereotype Content Model (SCM; Fiske et al., 2002) provides a framework for understanding these biases by modeling social cognition along two key dimensions: “warmth” and “competence.” Based on these dimensions, stereotypes can be categorized into four clusters: high competence and high warmth, high competence and low warmth, low competence and high warmth, and low competence and low warmth. Groups described to have high warmth and high competence are viewed in the most positive way, although their prevalence varies by cultural context—being more common in individualist societies but less pronounced in collectivist cultures (Fiske et al., 2002). In contrast, groups perceived as competent but low in warmth are subject to envious stereotypes, being viewed as proficient but not communal. Those viewed as low in competence but high in warmth face pitying stereotypes, seen as needing care and support from reference groups. Finally, groups portrayed as having both low warmth and low competence are seen in the most negative way, and are often subject to group derogation. Research suggests that the dimensions of warmth and competence are universal across culture (Cuddy et al., 2008), with multiple studies proving the effectiveness of the framework in analyzing stereotypes regarding various characteristics such as gender (Eckes, 2002), race (Sanders & Sullivan, 2010), disabilities (Canton et al., 2023), and age (Shimizu et al., 2024). Studies show that, according to the stereotype content model, older adults are placed in the high-warmth low-competence category (Cottrell & Neuberg, 2005; North & Fiske, 2013).

Beyond the SCM, new models for conceptualizing stereotype dimensions have also been proposed. The ABC model (Koch et al., 2016) introduces three dimensions: Agency, Beliefs, and Communion, where agency and communion correspond closely to the competence and warmth dimensions of the SCM. In addition, other literature suggests refining the SCM dimensions by subdividing warmth into sociability (or friendliness) and morality, and competence into ability and assertiveness (Abele et al., 2016).

The stereotype content model is a particularly valuable method when analyzing text with computational methods to identify stereotypes toward certain groups due to the development of word embeddings (Mikolov et al., 2013), which allows for vector representation of words while preserving their semantic meaning. This gave way to the development of the POLAR framework, which constructs dimension vectors for various concepts using antonym pairs, enabling the quantification of how certain words or phrases relate to the concept in question (Mathew et al., 2020). Previous literature has used psychological texts to create stereotype content dictionaries (Nicolas et al., 2021) and to extend them using automated methods (Qin & Tam, 2023). Since its conception, natural language processing methods have been developed to extend the unit of analysis to sentences, allowing quantitative analysis of the whole context in which the phrases are used (Reimers & Gurevych, 2019). Using these methods, researchers have started utilizing sentence embeddings to analyze stereotypes in various text data sources (Fraser et al., 2022, 2024).

## The current study

This study harnesses natural language processing (NLP) techniques to analyze text generated by OpenAI’s GPT-4o model, aiming to explore how the model’s semantic choices vary with age. The analysis is framed using the SCM (Fiske et al., 2002). This exploratory work contributes to two main aims: (a) Understanding how large language models generate text about older adults can inform stakeholders—such as policymakers and technology developers—who influence interactions between older adults and technology. Such insights can guide the development of policies or programs to address identified concerns; (b) adding a new dimension to the role of emerging technologies in the perpetuation of age-related stereotypes. This study seeks to address a critical gap in the readers’ understanding of how stereotypes may be embedded in responses to neutral, open-ended prompts.

## Research design and methods

### Data collection

GPT-4o is a multimodal generative AI model that can handle combinations of image, audio, video, and text input to generate content of the same form. Released to the public in May 2024, the model shows state-of-the-art capabilities in text generation (OpenAI, 2024). Text data collection was done through OpenAI’s GPT-4o API in February 2025. Rigorous pilot testing of prompts to collect text data was conducted to create a neutral prompt that did not fish for bias but elicited coherent responses from the model. The resulting prompt was of the structure “*Describe the personality of a [AGE]-year-old person.*” Responses to this prompt were collected with the AGE attribute ranging from 10 to 90 in increments of 10. The default parameter settings were as follows: temperature of 1.0, top_p value of 1.0, and a maximum output of 2,048 tokens. Here, the temperature parameter measures how deterministic or random the model’s output will be (Holtzman et al., 2019). A temperature value of 0.0 indicates that the model’s output becomes deterministic. Higher temperature values yield more stochastic outputs, and a high temperature value can lead to nonsensical outputs. This temperature value was chosen as previous studies have shown that a temperature value of up to 1.0 does not interfere with the quality of responses, allowing for a wide range of responses without losing coherence (e.g., Renze, 2024). The top_p parameter indicates the level of diversity in the responses to the prompt (Holtzman et al., 2019). A top_p value of 1.0, combined with a temperature setting of 1.0, enables the model to generate diverse responses (Ma et al., 2024). A token is the unit used in language models that represents a word or a part of a phrase. The parameter setting allows for a maximum of 2,048 tokens, or approximately 1,500 words. One hundred text responses were collected from the model for each age value, resulting in a total of 900 text samples. This sample size was derived from previous studies that compared human-authored text and AI-generated text, which extracted 100 samples of text for each dimension of analysis (Sardinha, 2024).

As sentence embeddings have lower accuracy for sentences that are three words or shorter (Shashavali et al., 2019; Wu et al., 2025), only sentences longer than three words were selected. Sentences that indicated the start of a list (i.e., ending with a colon, such as “Here are some common characteristics:”) were also filtered out. As most of the collected responses had opening and closing sentences at the start and end that did not describe the personality of the prompted age (e.g. “It’s important to note that personality can vary widely among individuals, regardless of age.”), the first and last sentences of each response were also excluded from analysis.

### Model selection and text similarity analysis by age

To analyze stereotype content in text samples describing individuals of different ages, this study employs a sentence embedding approach using the RoBERTa sentence transformer model (Liu et al., 2019). RoBERTa is a transformer-based language model that generates contextualized vector representations of text, where each sentence is mapped to a 1,024-dimensional embedding. These embeddings encode semantic and syntactic information, enabling the quantitative analysis of textual similarities and stereotype dimensions.

To compare descriptions of different age groups, sentence embeddings for all text samples were first generated using the RoBERTa model. To facilitate interpretability, dimensionality reduction was performed on the entire corpus using Principal Component Analysis. After reducing the dimensionality, the mean sentence embedding vector was computed for each text sample. To assess how similarly different age groups were described, cosine similarity was used to measure the distance between the mean sentence embeddings of different groups. Cosine similarity values close to 1 indicate high similarity in descriptions, while values closer to 0 suggest substantial differences in how different age groups are characterized (Reimers & Gurevych, 2019).

### Analysis of stereotype content dimensions using sentence embeddings

Following the methodology proposed by Fraser et al. (2022), stereotype content dimensions of “warmth” and “competence” were operationalized using sentence embeddings. Seed adjectives associated with warmth and competence were sourced from prior research on stereotype content (Nicolas et al., 2021). To generate reference sentences capturing these dimensions, adjectives were inserted into sentence templates:

- Single-adjective template: “These people are always [ADJECTIVE].”
- Two-adjective template: “These people are always [ADJECTIVE 1] and [ADJECTIVE 2].”

Sentence embeddings were generated for these stereotype content sentences using RoBERTa. These embeddings served as data to fit a Partial Least Squares (PLS) regression model, which maps sentence embeddings to stereotype content scores (Höskuldsson, 1988). The model generated warmth and competence values for input sentences on a scale from −1 to 1, where negative values indicated negative associations (e.g., cold or incompetent), and positive values indicated positive associations (e.g., warm or competent).

With the fitted PLS regression model, warmth and competence values were computed for each sentence in the collected text dataset. This resulted in a distribution of warmth and competence scores for descriptions of individuals across different age groups. The analysis of these distributions provided insight into how people’s personalities are described at different ages, revealing underlying patterns in age-related stereotypes within the text data.

### Word count analysis of stereotype content subdimension

To further examine the nature of text generated for different age groups, a word-level analysis was conducted. Nouns and adjectives were extracted from the text using the spaCy Python library and lemmatized to their base forms. To improve the reliability of the analysis, highly common words in each text sample were extracted with a method using Zipf’s Law of common terms (Saif et al., 2014). Words that appeared in more than six ages were excluded, as these were likely common phrases generated by the model rather than meaningful indicators of stereotype content. The full list of excluded words and the typical phrases they were used in is provided in Table S1 (see online supplementary material).

**Figure 1.**
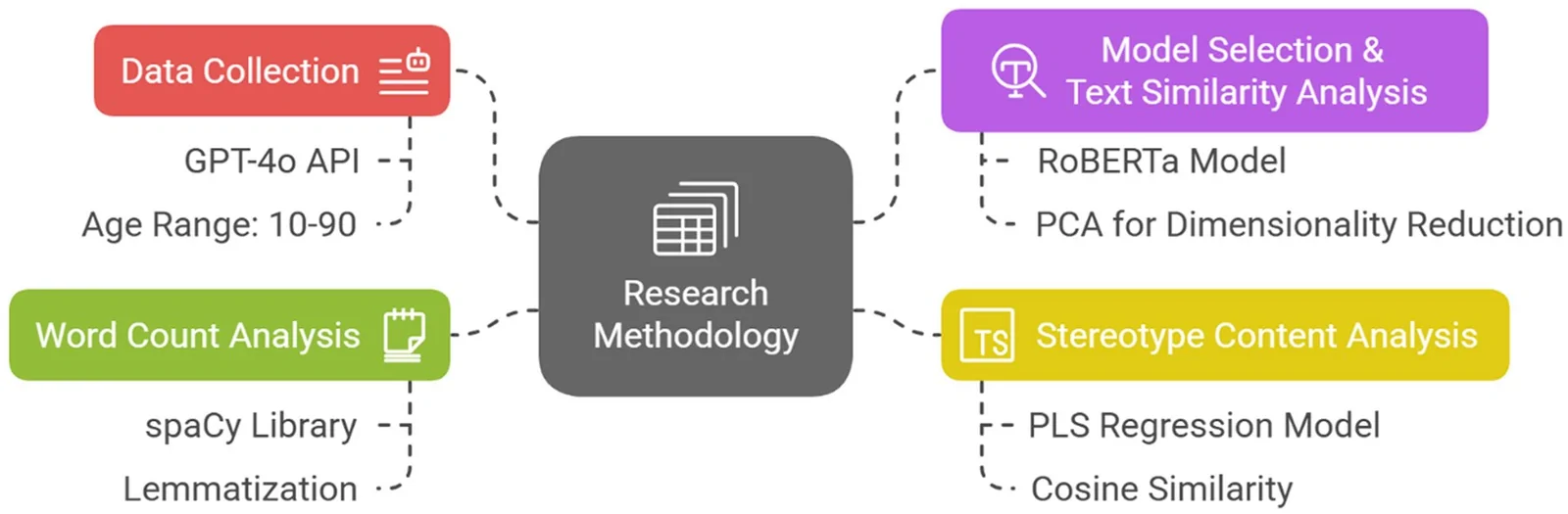
Flowchart of data collection and analysis methods.

Using the stereotype content lexicon provided by Nicolas et al. (2021), occurrences of words associated with positive and negative stereotype subdimensions were counted. The objective of the word count analysis is to further examine how the different text samples differ in content beyond calculated warmth and competence values. Thus, the subdimensions of warmth (or communion) and competence (or agency) are of interest, as these two dimensions are fundamental content dimensions present in many proposed stereotype models (Abele et al., 2016). Thus, this analysis focused on the subdimensions of warmth (sociability and morality) and competence (ability and assertiveness). While word count methods may be less effective than sentence-level analysis due to potential loss of context, such as negation or sarcasm (Fraser et al., 2022), this approach provides a complementary baseline for understanding how stereotype content varies across different subdimensions of the stereotype content model. A diagram outlining the data collection and analysis process is presented in Figure 1.

## Results

### Text similarity analysis by age

The cosine similarity heatmap of text embeddings between different age groups revealed three distinct clusters (Figure S1; see online supplementary material). Ages 10 and 20 form a group with moderate similarity (0.58) but little resemblance to other ages (−0.67 to 0.11). Ages 30, 40, and 50 are also part of a group with mild to moderate similarity (0.21–0.53). Older adults (ages 60, 70, 80, and 90) form the most cohesive group, with age 60 showing relatively moderate similarity to ages 70, 80, and 90 (0.35–0.53), while ages 70, 80, and 90 exhibit very high internal similarity (0.73–0.90). These findings suggest that textual descriptions of individuals generally fall into three broad clusters: a younger group (ages 10 and 20), a middle-aged group (ages 30, 40, and 50), and an older group (ages 60 and above), with particularly strong consistency among the oldest age ranges.

### Sentence-level modeling of stereotype content

**Figure 2.**
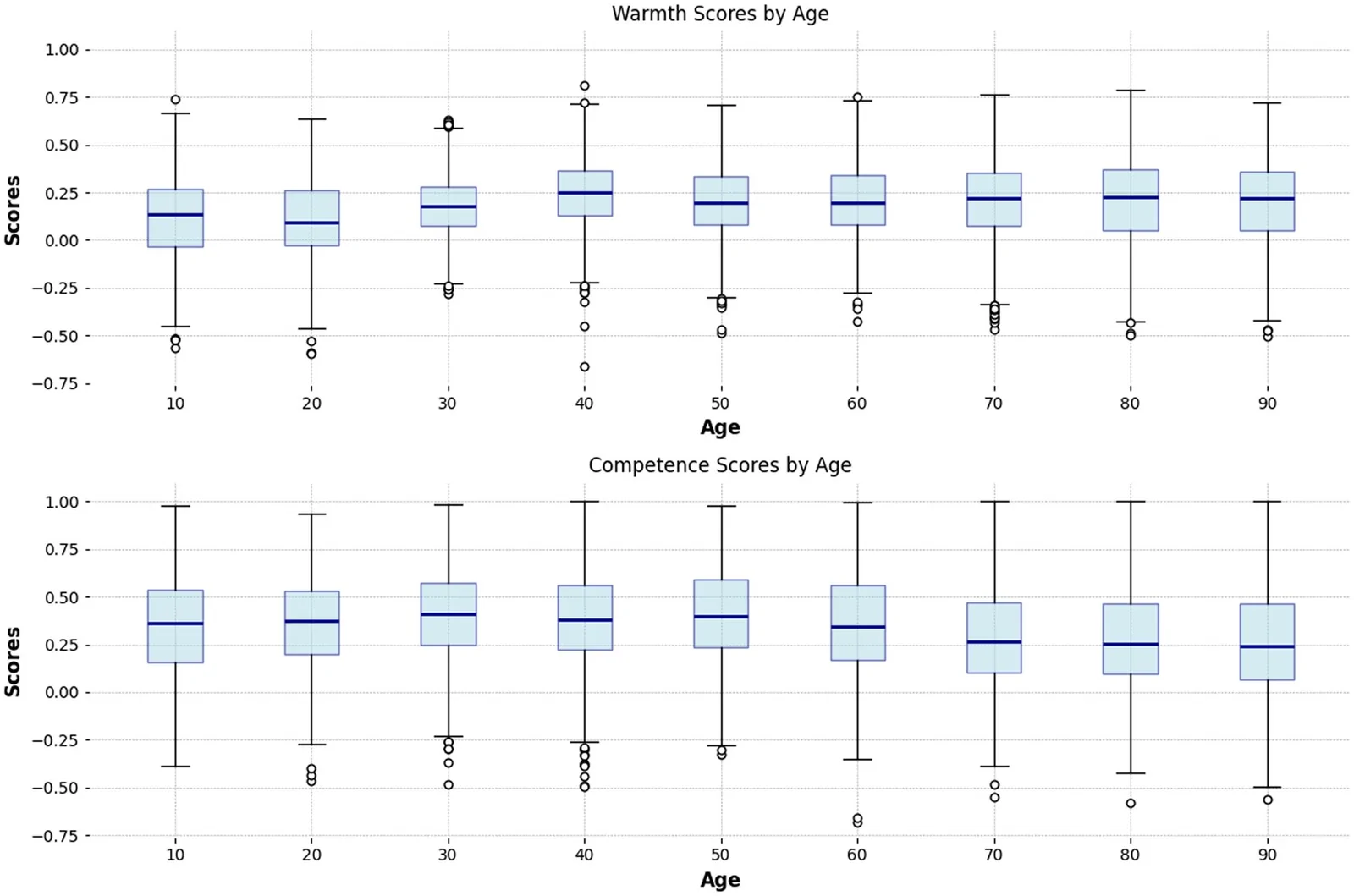
Box plot of warmth and competence scores by age.

**Figure 3.**
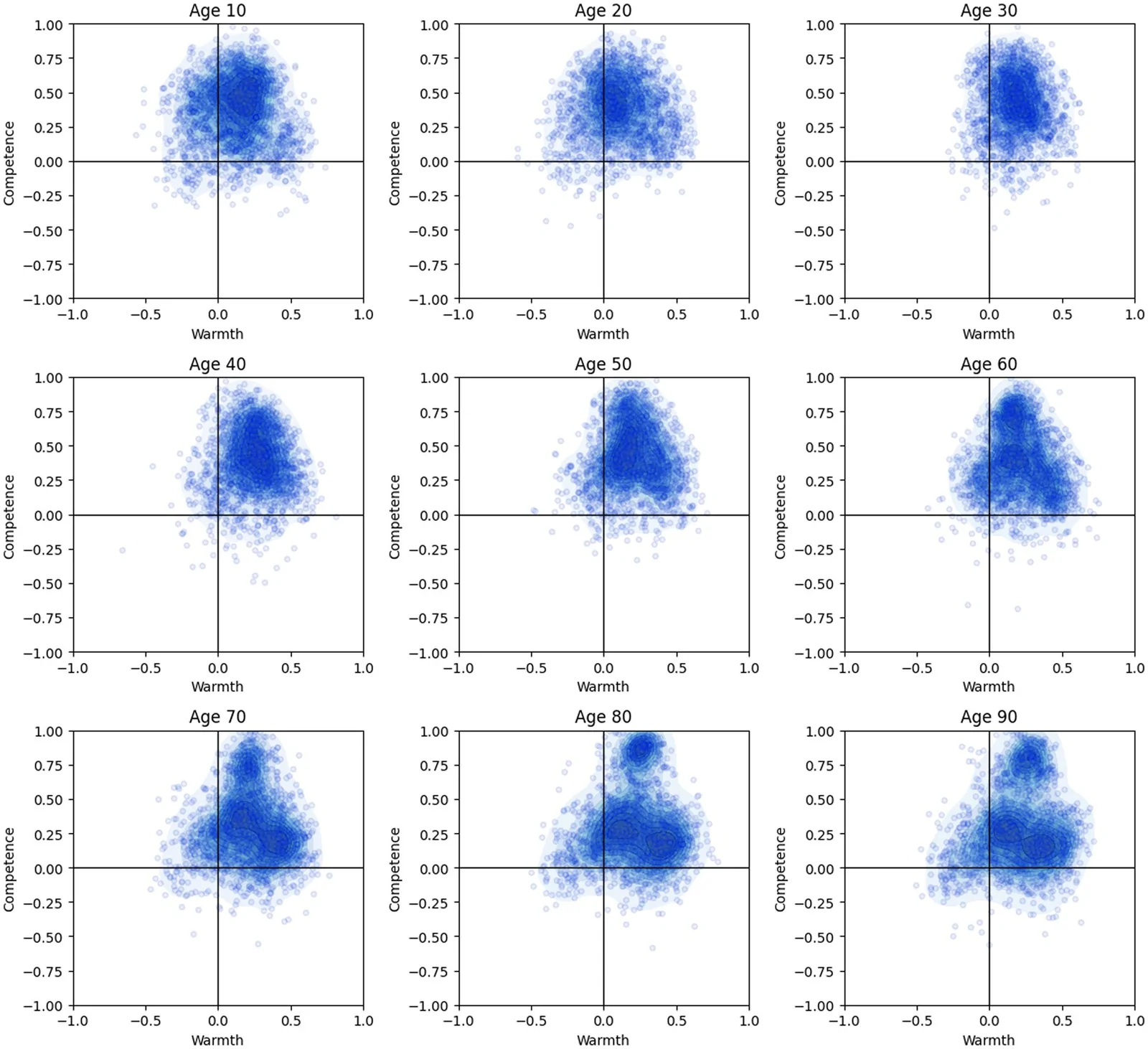
Scatter plots of warmth and competence distributions, in order of age from left to right, top to bottom.

Younger ages (10 and 20) had the lowest warmth scores and relatively high competence scores (see Figure 2). Warmth scores increased with age, peaking at age 40 with a mean value of 0.242 before slightly declining in older groups (Table S2; see online supplementary material). Competence scores were highest at age 30 with a mean value of 0.402 and remained relatively stable for ages 40 and 50 before declining steadily from age 60 onward. A scatter plot of the stereotype values by age is shown in Figure 3. The distribution of warmth and competence values varied significantly across age groups, as indicated by the results of Welch’s test: *F*(8, 5,662.3) = 67.68, *p* < .001 for the warmth dimension, and *F*(8, 5,667.8) = 38.50, *p* < .001 for the competence dimension. Welch’s test for equality of means confirmed that the differences in each stereotype dimension were statistically significant.

Typical example sentences for high and low values of each stereotype content dimension are shown in Table 1. Younger ages (ages 10 and 20) are seen as empathetic and adaptable, but emotionally volatile and easily frustrated in the warmth dimension. For competence, they are depicted as energetic and ambitious, but uncertain, anxious, and struggling with identity. Middle-aged individuals (ages 30–50) in the warmth dimension are described as compassionate and understanding, but they prioritize personal goals and practicality over idealism. Their competence is described as being confident and self-reliant, but at the expense of ambition for stability. Older adults (ages 60–90) are often characterized by warmth, generosity, and an appreciation for life’s simple joys, but may be perceived as stubborn due to ingrained beliefs. In terms of competence, they are described to be resilient and wise, but with less ambition and drive.

**Table 1.** Example sentences of stereotype content by different age groups.

Age group	Younger (10–20)	Middle-aged (30–50)	Older (60–90)
**High warmth**	“They start to develop a stronger sense of empathy and fairness, often showing concern for others and understanding different perspectives.” “This age group often demonstrates flexibility and openness to change, whether it involves career choices, living situations, or personal relationships.”	“Many individuals become more compassionate and patient.” “With years of social interaction and life lessons, many have developed a strong sense of empathy and understanding.”	“There can be a profound appreciation for the simple joys in life, such as nature, family, and shared meals.” “They often display generosity, whether in terms of sharing their time, resources, or knowledge with others.”
**Low warmth**	“They can be emotionally volatile, with individuals experiencing intense emotions as they navigate relationships, careers, and their future.” “They may become easily frustrated or upset, especially when facing disappointment or challenges.”	“There’s often less concern about others’ opinions and more focus on personal goals.” “They may prefer solutions that are realistic and grounded over idealistic but impractical ideas.”	“Some become more cautious with age, perhaps due to physical limitations or a heightened awareness of their mortality and the passage of time.” “On the flip side, some older individuals might exhibit stubbornness due to deeply ingrained habits and beliefs.”
**High competence**	“This age group often possesses high energy levels and the physical endurance to take on various activities and challenges.” “They may have ambitious goals and a strong belief in their ability to achieve them.”	“They’ve developed the ability to rely on themselves and make independent decisions.” “This self-awareness often translates into greater confidence, as they have spent years honing their skills and understanding what they want out of life.”	“Having faced various ups and downs, people at this age are usually resilient and better equipped to handle life’s challenges.” “Having lived through a wide range of life events, they often possess a wealth of knowledge and wisdom.”
**Low competence**	“It’s not uncommon for individuals in this age group to feel uncertainty about their future, leading to stress or anxiety as they make important life decisions.” “At this age, many people are still figuring out their identity and what they want out of life.”	“There’s often a desire for more stability in life, whether that’s in their career, relationships, or living situation.” “Career ambitions might plateau or shift toward seeking a better balance between professional commitments and personal life, often valuing time and experiences over material gain.”	“Priorities might shift toward valuing relationships and experiences over material possessions or career ambitions.” “Many have learned not to take themselves too seriously and to approach the world with a more laid-back attitude.”

### Word count analysis of stereotype content subdimensions

Table 2 presents the complete word count data for stereotype content subdimensions. Across all ages, negative terms for all subdimensions were rare, mostly accounting for less than 1% of the total word count. Positive ability terms were the most frequent, comprising over 8% of the total words across all age groups and remaining relatively stable across different age groups. On the other hand, positive assertiveness terms exhibited a sharp decline with increasing age. Figure S2 (see online supplementary material) provides bar plots showing the number of stereotype content words for each subdimension (positive and negative).

**Table 2.** Number of stereotype content words for each sub-dimension (positive/negative).

Age	Total word count	Warmth				Competence			
		Sociability		Morality		Ability		Assertiveness	
		Positive	Negative	Positive	Negative	Positive	Negative	Positive	Negative
**10**	13,075	1,092	35	436	269	1,118	20	1,261	44
**20**	14,630	608	33	164	26	888	0	1,021	135
**30**	14,903	541	8	359	8	1,120	1	1,049	79
**40**	14,795	572	9	420	8	1,343	5	940	81
**50**	14,583	583	6	313	14	1,561	8	890	70
**60**	15,077	603	9	377	8	1,555	20	727	56
**70**	14,910	668	24	462	20	1,381	55	524	73
**80**	14,502	632	34	420	21	1,335	82	418	109
**90**	14,264	598	28	367	39	1,218	111	398	135

Age 10 stood out with a notably high proportion of positive sociability terms and negative morality terms. Further analysis revealed that both were influenced by frequent mentions of children engaging in “play,” a word that was sometimes categorized as a negative morality term due to one of its meanings being synonymous with “deceive.” In addition, negative morality terms in age 10 texts often appeared in discussions about children learning concepts of right and wrong.

## Discussion

The findings from the results section highlight several important observations regarding age-related stereotypes in language models. First, the similarity analysis suggests that descriptions of older adults, aged 60 and above, are more homogenized compared to those of younger age groups. As Judd and Park (1988) described the homogenization of out-groups as a basis for the formation of stereotypes, the high consistency in descriptions of older adults suggests that stereotypes about older adults are embedded in the language model.

The distribution of stereotype contents across different age groups reveals that, while descriptions generally exhibit positive warmth and competence, notable differences exist by age. Older adults tend to receive higher warmth scores and lower competence scores compared to younger individuals. A deeper analysis of the generated text indicates that older adults are frequently described as generous, caring, and wise, yet al.o as having fewer goals, being set in their ways, and displaying stubbornness. These portrayals are consistent with previous media representations of older individuals in films, television series, and social networking services (Lauzen & Dozier, 2005; Levy et al., 2014; Markov & Yoon, 2021; Robinson et al., 2007).

A particularly concerning trend is the relative lack of assertiveness in descriptions of older adults. Given the increasing integration of LLMs into AI-powered virtual assistants and automated decision-making systems—such as hiring algorithms, loan approval systems, and healthcare triage tools—such portrayals may contribute to the reinforcement of stereotypes in user interactions. Repeated exposure to these descriptions can lead to the internalization of such stereotypes, potentially influencing how older individuals perceive their own agency and capabilities (Marquet et al., 2019; Tresh et al., 2019).

The reproduction of stereotypical narratives about older adults raises critical concerns. When embedded in AI systems, these depictions risk reinforcing structural ageism that shapes societal expectations about aging. Research has shown that even stereotypes that are framed positively can elicit negative emotional responses (Czopp, 2008; Siy & Cheryan, 2016). Moreover, positive stereotypes are especially problematic in that they are less likely to trigger emotional vigilance, yet still reinforce potentially harmful assumptions (Kay et al., 2013). As these narratives become embedded in AI-generated content, they may be internalized by older individuals, diminishing their sense of agency and potentially contributing to declines in cognitive and psychological performance (Hehman & Bugental, 2015; Hess et al., 2003). Over time, this dynamic can contribute to a cycle of injustice in which older adults are excluded from full digital participation and representation.

Portrayals that depict older users as less proactive can skew the design and distribution of technology, further deepening the digital divide (Chu et al., 2022). From a usability perspective, these biases can undermine the effectiveness and accessibility of AI systems for older users. When unchecked, language models trained on age-stereotypical data may generate patronizing or infantilizing responses, mimicking forms of elderspeak. In human caregiving contexts, elderspeak has been shown to increase resistance to care and reduce the engagement of older adults (Zhang et al., 2020). When replicated by AI assistants, such communication patterns may alienate users, diminish trust in the system, and reduce the willingness of older adults to engage with digital tools.

### Limitations

While this study presents a novel method for quantitatively assessing age stereotypes in AI-generated text, several limitations should be acknowledged. First, the prompt used to collect text samples was intentionally designed to be neutral and to avoid eliciting bias. While this approach supports consistency across outputs, it does not fully capture the diversity of prompts that large language models encounter in real-world applications. Considering that the language and framing of a prompt can influence the content of generated text, additional research is needed to examine how the age-related stereotypes identified here may emerge under more varied or naturalistic prompting conditions. Second, the AI model used in this study, OpenAI’s GPT-4o, represents a single snapshot in time. Large language models are periodically updated and retrained, which can alter their underlying representations and outputs. As a result, the patterns of positively framed age stereotypes observed in this analysis may not remain stable as newer versions of the model are released. Ongoing evaluation across different model updates will be necessary to understand the persistence or changes in these stereotypes. Finally, this research focused exclusively on textual prompts and textual outputs. Generative AI systems are rapidly expanding to include multimodal capabilities such as image generation, audio synthesis, and cross-modal interactions. These emerging modalities may carry age-related stereotypes in forms that differ from texts alone. Future studies should extend this work to include multimodal inputs and outputs in order to develop a more comprehensive understanding of how age stereotypes manifest across different types of AI-generated content.

## Conclusion and implications

Existing research has demonstrated that age stereotypes contribute to the discrimination against certain groups, and thus are generally harmful to society (Allen, 2016; Levy, 2009). This study examined whether LLMs generate content that contains age stereotypes when responding to open-ended prompts designed to mimic realistic user interactions. GPT-4o, along with its lightweight variant GPT-4o-mini, was selected for analysis due to its prominence as the foundation of OpenAI’s ChatGPT, currently one of the most influential language model interfaces.

The findings of this study indicate that while GPT-4o’s generated descriptions of older adults were framed positively, they nonetheless exhibited stereotypical representations. These stereotypes have the potential to elicit adverse emotional reactions from older adult users. This underscores a crucial point: The absence of explicitly negative language does not necessarily ensure a positive or unbiased user experience. The presence of subtle stereotypes may still influence perceptions and interactions, warranting further examination. This finding provides a conceptual foundation for datasets and frameworks to assess and compare large language models’ ability to mitigate the spread of social bias.

Given these findings, further research is needed to examine the psychological and social impacts of such descriptions on older adults. Investigating user reactions and potential harms will be essential in assessing the broader implications of language model outputs in real-world applications. To build on the insights from this study, future research should also expand the scope of analysis beyond a single LLM provider. Comparative evaluations across generative models, including Google’s Gemini, Meta’s LLaMA, Mistral AI, Claude, and DeepSeek, would allow for a more generalizable understanding of how age stereotypes are embedded across different architectures, training data sources, and design approaches. Such cross-model comparisons could help determine whether these biases are systemic or model-specific, and guide the development of more robust and inclusive language technologies.

To address the societal risks posed by biased model outputs, this study emphasizes the need for policy interventions aimed at mitigating bias in AI-generated content. One policy recommendation is to develop community-defined annotation and bias mitigation guidelines, allowing diverse perspectives to voice concerns that might otherwise be overlooked. This would help ensure that LLMs produce more equitable and inclusive representations. In addition, as expecting perfectly neutral LLMs is unrealistic, evidence-based social discussions and consensus on acceptable levels of bias are needed, with oversight from stakeholders across the private, civil, and public sectors. Furthermore, there is a need for collaboration with diverse stakeholders, including older adults—an increasing segment of LLM users—in the design and refinement of LLMs and AI-driven agents.

## Supplementary Material

gnaf291_Supplementary_Data

## Data Availability

Raw data were generated using OpenAI’s GPT 4o Model. Derived data supporting the findings of this study are available from the first author, WH, on request.
