# Supplementary material for: An exploratory semantic analysis of age-related stereotypes in OpenAI’s GPT 4o model
Source: Gerontologist. 2025 Dec 8;66(2):gnaf291. doi: 10.1093/geront/gnaf291 (PMC13148887; doi:10.1093/geront/gnaf291)

**Supplementary Material Table** **1.**

*Words Excluded from Word Count Analysis*

| Categories | Details |
| --- | --- |
| Words used for mentioning of age and age groups | 'year', 'age', 'old(s)', 'people', 'person', ‘life’ |
| Common words that occur as a response to the prompt | 'many', 'personality', ‘trait(s)’, 'personal', 'experience(s)', ‘individual(s)’, 'relationship(s)', 'sense', ‘important’ |
| Phrases used to describe people | 'self', 'other(s)' |

* Note: A total of 16 different words (not counting plural forms) were excluded from analysis.

**Supplementary Material**  **Figure 1.**

*Heatmap of Cosine Similarity Between Generated Texts by Age, After Dimensionality Reduction.*

**
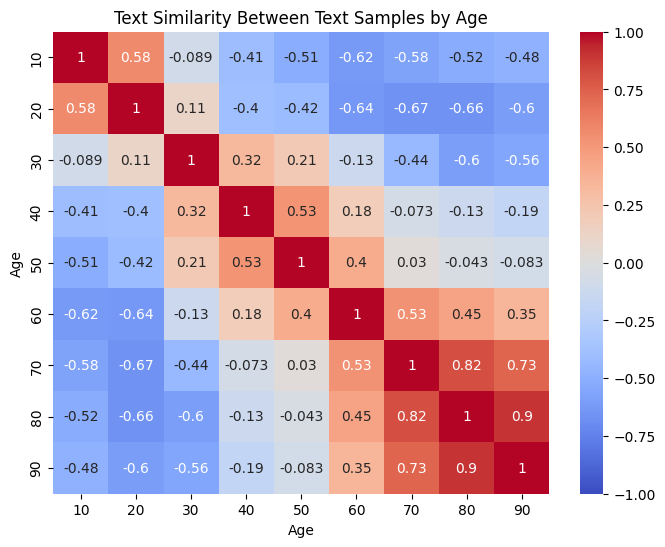
**

**Supplementary Material** **Table 2.**

*Descriptive Statistics of Warmth and Competence Values of Generated Text for Each Age*

| **Age** | **Sentence Count** | **Warmth** | | **Competence** | |
| --- | --- | --- | --- | --- | --- |
|  |  | **Mean** | **SD** | **Mean** | **SD** |
| 10 | 1561 | 0.119 | 0.218 | 0.345 | 0.252 |
| 20 | 1495 | 0.109 | 0.217 | 0.363 | 0.233 |
| 30 | 1567 | 0.178 | 0.159 | 0.402 | 0.231 |
| 40 | 1573 | 0.242 | 0.178 | 0.381 | 0.243 |
| 50 | 1534 | 0.202 | 0.187 | 0.405 | 0.246 |
| 60 | 1546 | 0.206 | 0.191 | 0.363 | 0.258 |
| 70 | 1515 | 0.208 | 0.216 | 0.300 | 0.278 |
| 80 | 1470 | 0.200 | 0.231 | 0.310 | 0.301 |
| 90 | 1398 | 0.194 | 0.232 | 0.290 | 0.305 |

**Supplementary Material Figure 2.**

*Number of Stereotype Content Words for Each Sub-Dimension (Positive/Negative).*


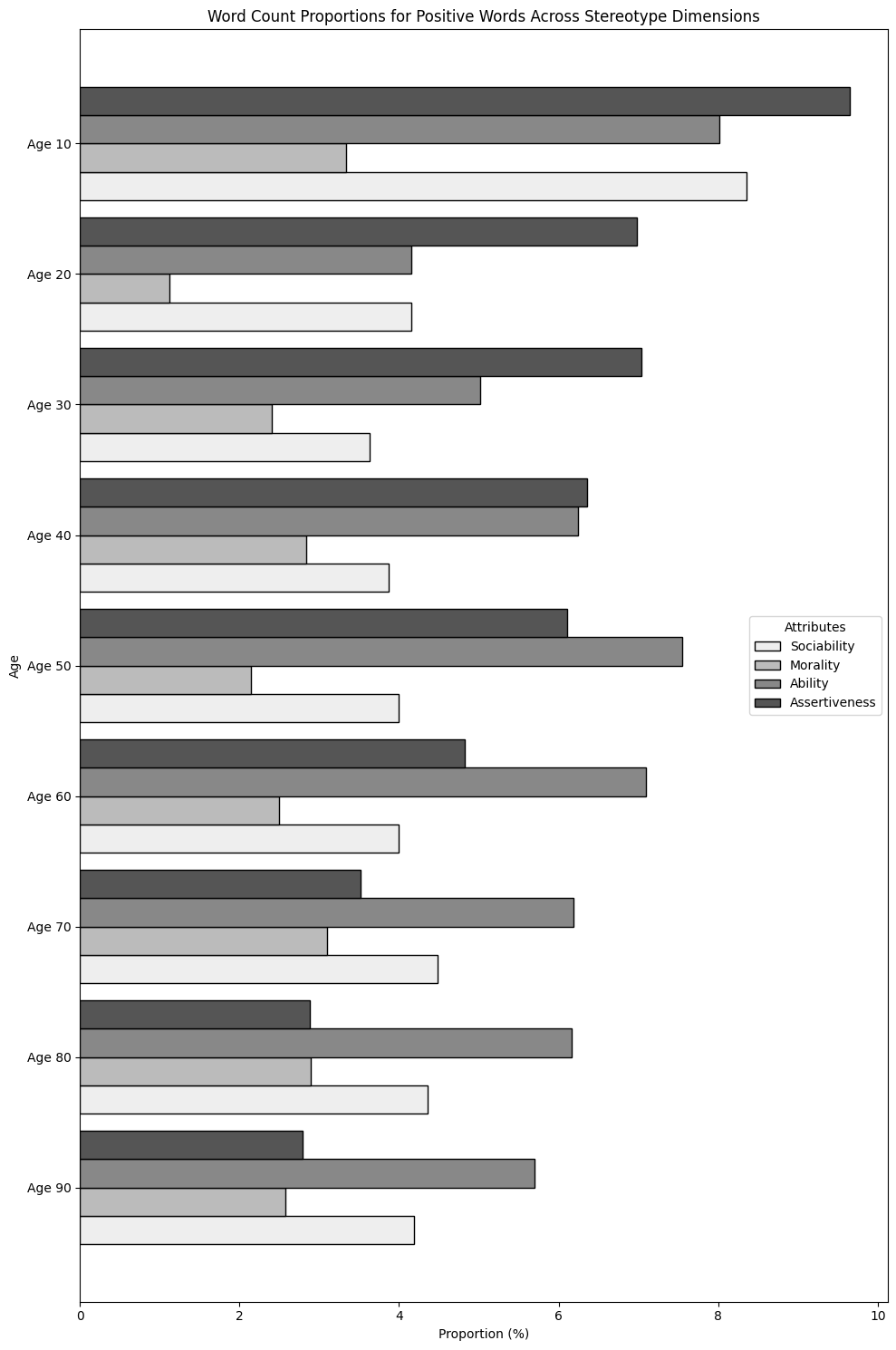


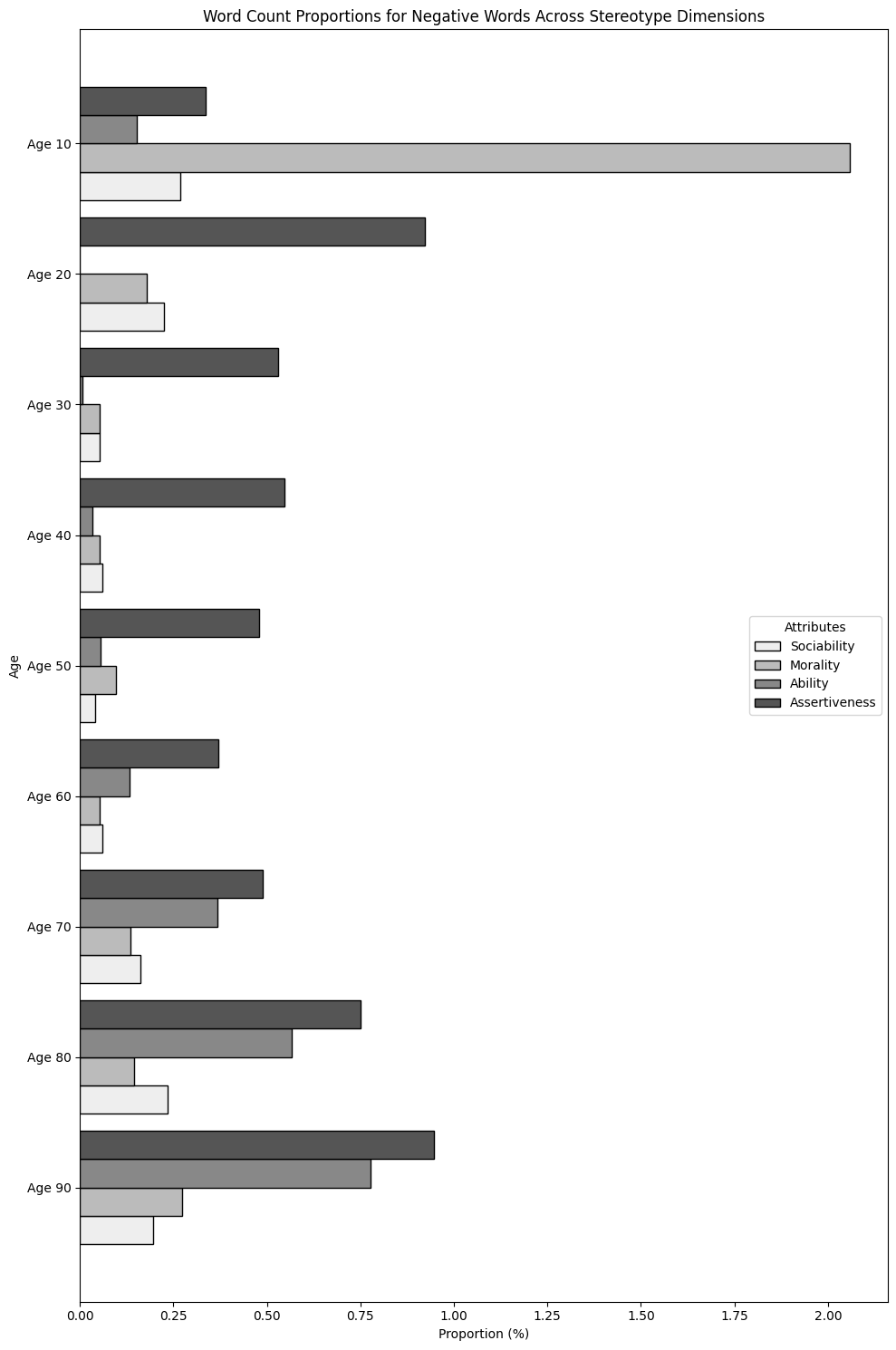

Supplement: gnaf291_Supplementary_Data [file gnaf291_supplementary_data.zip › Hong & Choi Suppl.docx]
